# Escaping from poverty trap: a choice between government transfer payments and public services

**DOI:** 10.1186/s41256-017-0035-x

**Published:** 2017-06-05

**Authors:** Sixia Chen, Jianjun Li, Shengfeng Lu, Bo Xiong

**Affiliations:** 1 School of Public Finance and Taxation, Zhongnan University of Economics and Law, Wuhan, Hubei China; 2 School of Public Finance and Taxation, Southwestern University of Finance and Economics, Chengdu, Sichuan China; 3 Economics and Management School, Wuhan University, Wuhan, Hubei China

**Keywords:** Government transfer payments, Public services, Anti-poverty, Crowd-out effect, Inductive effect

## Abstract

**Background:**

Anti-poverty has always been an important issue to be settled. What policies should be selected to help individuals escaping from the poverty trap: by directly offering transfer payments or indirectly providing public services? This paper is among the first to explore the effects of public anti-poverty programs system in China.

**Methods:**

We Using unbalanced panel data of China Health and Nutrition Survey (CHNS) from 1989 to 2009, we demonstrate how the individual poverty status is determined through a four-staged simultaneous model. We choose the 3SLS (Three Staged Linear Squared) methodology to do the estimation.

**Results:**

GTPs (Government Transfer Payments) don’t have positive effects on poverty reductions. The results demonstrate that GTPs increasing by 10% makes private transfer payments decrease by 3.9%. Meanwhile, GTPs increasing by 10% makes the household income decreased by 27.1%. However, public services (such as medical insurance, health services, hygiene protection etc.) have significantly positive impacts on poverty reduction. Public services share a part of living cost of the poor, and are conducive for people to gain higher household income.

**Conclusions:**

GTPs given by governments are not effective in reducing the poverty, as a result of “crowd-out effect” and “inductive effect”. However, public services are suggested to be adopted by governments to help the poor out of the poverty trap.

## Background

China has seen a fast economic growth over the past thirty years. However, there are still a number of people suffering from poverty. Since the mid-1980s, Chinese government has implemented a series of public policies to fight against poverty, including heavy investment on anti-poverty programs. According to China Statistical Bureau, the poverty rate in China has fallen from 30.7 to 1.7%. While the absolute poverty population, defined as people who are below the national standard poverty line, drops from 250 million to 14.78 million from 1978 to 2007.

However, the anti-poverty work becomes more and more difficult to step forward. For instance, the poverty reduction rate was roughly about 1.5% each year from 1978 to 1999, but the rate has been held up around 0.26% for the following eight years. Moreover, some new problems, known as “temporary poverty” and “non poverty-back-to-poverty” raise up. They somehow challenged the sustainability of the on-going anti-poverty work. For example, a large number of people are migrating from rural areas into cities nowadays, and they may become the potential poor as a result of higher living cost in cities.

The fiscal budget is stringent and fiscal resources are limited. Therefore, it is of key importance to explore what are the most effective policies to help the low-income people out of poverty trap. Generally speaking, there are three main tools to reduce the poverty rate. The first is known as government transfer payments(GTPs). By using this policy, the government sets the standard to distinguish target groups and directly offer cash funds to them. There are kinds of government transfer payments in China. They include but are not limited to public grants to the disabilities,^1^ cash transfers to people whose spouse(or parents) dies(die) as a result of working, subsidies to people whose total earnings is below the national minimum living cost^2^ etc. Overall, GTPs means direct cash funds offered by governments to the poor who need financial help.

However, governments can also help the poor by providing primary public services, such as education, health services, sanitation etc. As it doesn’t mean government offering cash to the specific group directly, public services can be seen as an indirect way for governments to help people out of poverty. Basically, public services do not target any special group, i.e., residents have equal access to public services once they are provided. The cost of the services (or goods) are mainly undertaken by governments instead of individuals. Although primary public services may not reach the best qualities, they essentially meet the basic demand of people. All of the residents including the poor are eligible to have free access to utilize these services or they spend just a few user fees in gaining the usage.

The last financial resource the poverty could obtain is known as private transfer payments(PTPs). They could be offered by their employers, or relatives, or friends. This kind of transfer payments can be in the form of cash or in-kind benefits.

According to the previous literature, government transfer payments are often justified by their presumed effects on poverty reduction, as GTPs seem to increase the income of low-income group. However, the empirical evidence are ambiguous and inconclusive. By using the country-level data, a strand of literature find that public transfer payments can significantly reduce poverty [1–3]. Some relevant studies also highlight the importance of GTPs in anti-poverty system for the low-income residents in rural China, who are characterized as the “absolute poverty”[4]. Du & Park [5] believe that the governmental transfer system in urban China can effectively target the poor and help them out of the poverty trap. Wu and Ramesh [6] empirically study poverty-reduction effects of the Minimum Living Standard Assistance Program in China. They find that governments directly giving money to the poor is proved to be an effective tool for poverty reduction [6]. Similar findings are also provided by other studies [7, 8].

However, some studies come to opposite conclusions. They find GTPs don’t have positive effects on reducing poverty [9–11]. More studies offer explanations for the failure of GTPs. Basically, there are two major problems associated with this policy. One is known as “funds misappropriation”. In reality, GTPs are initially allocated by the central government, but implemented by prefecture governments(or cities for brevity). In many cases, without strict supervisions, city governments have strong inclinations to divert these funds to support local economic growth rather than helping the poor. Therefore, the lower-level governments, such as counties and townships, usually find themselves lack of money to offer subsidies to the poor. The other one is called “targeting errors”. In other words, there are probabilities that high-income level people are mistakenly regarded as the poor who are in need of financial help [12, 13]. Xia et al. [14] apply with Chinese Household Income Project(CHIP) data set and find that anti-poverty policies have little effect on reducing urban poverty in China. Avram [15] examines in a comparative setting the role social assistance plays in reducing income poverty in eight Central and East European countries. Wang & van Vliet [16] explores the developments of social assistance and minimum income benefits across 14 Western European countries, 12 Central and Eastern European countries and 7 non-European countries. They find that the effects of governmental direct assistance on poverty reduction depend on institutional environment conditionally.

From another perspective, some literature prove that public services could reduce the poverty by increasing people’s earning capacity [17]. Some studies find that public expenditures on education and medical services have positive impacts on reducing poverty [18–21]. Strauss & Thomas [22], Quisumbing [23] discover that health insurance plays an important role in helping people out of poverty. Other public services, such as water supply, sewage disposal services as well as electric power facilities, are proved to effectively improve the living standard of low-income people [10, 24]. However, there are exceptions. Castro-Leal et al. [25] examines the impact of public spending on education and health care in several African countries. They find these programs are not pro-poor [25]. Wagstaff et al. [26] estimate the pro-poorness index of government health expenditure across 69 countries. They also find government health expenditures are pro-rich [26]. Similar evidences are provided by some other studies [27–29].

Besides GTPs and public services, private transfer payments(PTPs) can also help the poverty. As other individuals directly offer cash or in-kind benefits, it enhances the total income of the poor. However, some literature find out the crowd-out effect between GTPs and PTPs. Increasing the amount of GTPs(PTPs) would be likely to reduce the amount of the other one [30, 31].

As far as we have discussed, the impact of GTPs (public services as well) has not come to consensus. The most important reason lies in the endogeneity concerns. For example, whether GTPs result from the poverty status or whether poverty status results from GTPs is unclear. Both of these cause-and-effects relationships are possible, suggesting that GTPs and the individual poverty status are likely to be jointly endogenously determined. Moreover, since there may exist the crowd-out effect between GTPs and PTPs, PTPs may also be determined simultaneously as soon as GTPs approach the equilibrium point.

To account for this potential simultaneity, as well as examine interrelationships among GTPs, PTPs and public services, we estimate the empirical relationship between GTPs and poverty status using the simultaneous equation models(SEM). At the same time, we introduce four determination equations into SEM models: the household income, GTP, PTP and poverty status. And we use the three-staged least squares method to do the estimation. By applying with this methodology, we could not only overcome endogeneity concerns, but also demonstrate interrelationships among these three anti-poverty tools.

In this paper, we aim to do several tests with panel data from China Health and Nutrition Survey (CHNS). Firstly, how do GTPs, PTPs and public services affect the poverty status respectively, after accounting for endogeneity concerns. Secondly, how would GTPs, PTPs and public services affect each other. Thirdly, we check for heterogeneous effects.

This paper contributes to the extant literature as follows: Firstly, to the best of our knowledge, this is the first paper to study the effect of governmental transfer payments on poverty reductions in China. By solving the endogeneity concerns, it justifies the “crowd-out effect” as well as the “inductive effect” of GTPs. Secondly, SEM is applied to reveal interrelationships among GTPs, PTPs and public services. We overcome endogeneity concerns raised by using a single equation. Finally, our paper enriches the literature by providing empirical evidence at household-level.

The rest of paper is organized as follows: Section 2 introduces the identification framework and data, empirical results are presented in section3, followed by section 4 to further discuss the mechanisms and do robustness checks, and Section 5 is to conclude.

## Methods

### Identification framework

As aforementioned, SEM suits rightly to estimate the impact of GTPs on the poverty status, as well as explore interrelationships among three anti-poverty policies. Our estimation model can be expressed as follows:

1 $$ {Y}^h={f}_1\left({c_1}^h,{c_2}^h,{z_1}^h,{g}^h;{\theta}_1,{\theta}_2\right)+{\mu_1}^h $$

2 $$ {P}^h={f}_2\left({Y}^h,{c_1}^h,{z_2}^h;{\theta}_1,{\theta}_2\right)+{\mu_2}^h $$

3 $$ {T}^h={f}_3\left({Y}^h,{P}^h,{c_1}^h,{c_2}^h,{z_3}^h;{\theta}_1,{\theta}_2\right)+{u_3}^h $$

4 $$ Povert{y}^h={f}_4\left({Y}^h,{P}^h,{T}^h,{c_1}^h,{g}^h;{\theta}_1,{\theta}_2\right)+{u_4}^h $$

Where:

*h =* variables at household-level,

*Y* ^*h*^ = household income,

_*P*_^*h*^ = government transfer payments,

_*T*_^*h*^ = private transfer payments,

_*Poverty*_^*h*^ = poverty status at household-level,

_*g*_^*h*^ = public services,

_*c1*_^*h*^ = demographic characteristics at householder-level,

_*c2*_^*h*^ = working types at householder-level,

_*z1*_^*h*^ = education level of the householder,

_*z2*_^*h*^ = a set of policy-dependent characteristics at household-level,

*z*_*3*_^*h*^ = assets at household-level,

*θ*_*1*_ = year dummies,

*θ*_*2*_ = county dummies,

*u* ^*h*^ = random errors.

Our estimation model is partially based on Maitra & Ray [2]. The whole estimation system includes four equations. Equation (1) specifies the household income, as a function of four exogenous variables: demographic characteristics, householder’s working type, householder’s educational level and public services. Equation (2) specifies the government transfer payments as a function of the household income and two exogenous variables, including demographic characteristics and policy-dependent characteristics. Equation (3)specifies private transfer payments as a function of two endogenous variables(household income and GTPs), and three exogenous variables(demographic characteristics, working types and family assets). Equation (4) specifies the poverty status as a function of three endogenous variables(household income, GTPs, PTPs), and two exogenous variables(demographic characteristics and public services). Overall, household income, GTPs, PTPs and the poverty status are endogenous variables and determined simultaneously. While public services, as well as other controlling variables, are exogenous variables and predetermined.

Methodologically, as four variables are jointly determined and error terms of these four models may be correlated, OLS is not appropriate to estimate the model. Usually, two-staged least squares(2SLS) and three-staged least squares (3SLS) are mostly used. By adopting 2SLS method, it means that we regress household income on all the exogenous variables in Equation (1) (for an example), and then we estimate the fitted value of household income. In the next step, we use the fitted value of household income as an IV variable into Equation (2), *et cetera*. This could yield consistent estimates of parameters because the fitted value of the first stage is uncorrelated with the error term in the next stage regression. However, heteroscedasticity may still exist among our estimators of four different models. While by using 3SLS, we eliminate the heteroscedasticity concerns by using GLS estimation after the 2SLS method.

We also control for year fixed effects and county fixed effect in each equation. By satisfying both the rank condition and the order condition, SEM system can be only specified. We have several robust checks by changing our systematic models to various specifications and including different variables. However, this doesn’t change our results much.

### Data and variables

#### Data sources

The data we use come from “China Health and Nutrition Survey” (CHNS), implemented collaboratively by the Carolina Population Center at the University of North Carolina at Chapel Hill and the National Institute for Nutrition and Food Safety at the Chinese Center. This survey aims to provide data for study of disease control and prevention. And CHNS database is widely used for studies of poverty, health and income inequality [32–36].

During 1989–2009 year period, eight waves of household surveys are held annually. The database draws the sample restricted roughly to 4400 households with 19000 individuals from nine provinces that are broadly representative of China’s rich regional variation. The provinces include *Liaoning*, *Heilongjiang*, *Jiangsu*, *Shandong*, *Henan*, *Hubei*, *Hunan*, *Guangxi* and *Guizhou. Liaoning* and *Heilongjiang* is heavy industry provinces in the northeast; *Jiangsu* and *Shandong* are dynamic high growth provinces in China’s east coastal regions; *Henan*, *Hubei* and *Hunan* are less developed provinces in the middle of China; *Guangxi* and *Guizhou* are much less developed ones in the west of China.

There are also several caveats to mention. Basically, the questioned households are roughly the same for each wave of survey. However, new households enter or extant households exit our sample each wave. About 6.9% of the sample, on average, is refreshed randomly each wave of the survey. Thus, the panel data is not balanced. In addition, community questionnaire is also implemented at commune-level, from which we are able to obtain data of public services.

Two data sets, including both “household survey” and the “adult survey”, are drawn from CHNS. For the former one, it provides detailed information about the entire household. They include but are not limited to total household income, income sources, family background and other characteristics at household-level. However, until now, we are not able to obtain any information for householders. To control for characteristics at householder-level, we match “household survey” with the “adult survey” through year and ID code.^3^ “Adult survey” data set provides details of adults who are over 18 years old, such as demographic status, occupations, *etc*. It also asks every respondent if he(she) is the householder of his(her) family. Therefore, we have information of householders.

Sample distribution and variable statistics are presented in Table 1. As shown in Table 1, for each survey year, we have about 4,000 households. The sample is proportionally distributed among nine provinces, each province has about 11% of total households. Four waves of the survey, all of which are after 2000, account for 55% of total sample. This ensures representativeness of our sample to reflect present circumstances. Households from urban areas are significantly less than that from rural areas. Rural households are twice as big as urban sample. Multi-children families constitute almost over 7% of total observations in each province, whereas “one-child” family is about 3% comparatively.

**Table 1:** Descriptive statistics

Province/Year	Obs	Proportion (%)	Distribution of Urban/Rural		Whether one-child household	
			Urban (%)	Rural (%)	Yes (%)	No (%)
Liaoning	3221	10.02	3.3	6.72	2.49	7.52
Heilongjiang	2386	7.42	2.51	4.91	2.09	5.33
Jiangsu	3731	11.61	3.57	8.04	3.72	7.89
Shandong	3689	11.48	3.7	7.78	3.18	8.3
Henan	3774	11.74	3.75	7.99	3.08	8.66
Hubei	3760	11.7	3.85	7.85	2.81	8.89
Hunan	3747	11.66	3.87	7.78	2.88	8.77
Guangxi	3897	12.12	3.97	8.15	2.61	9.51
Guizhou	3942	12.26	3.92	8.34	3.04	9.22
1989	3791	11.79	3.85	7.94	2.72	9.07
1991	3607	11.22	3.59	7.63	2.83	8.39
1993	3428	10.66	3.26	7.4	2.59	8.08
1997	3838	11.94	3.96	7.98	3.28	8.66
2000	4329	13.47	4.42	9.05	3.41	10.05
2004	4339	13.5	4.45	9.04	3.5	9.99
2006	4374	13.61	4.44	9.17	3.72	9.88
2009	4441	13.81	4.47	9.35	3.85	9.97
Total	32147	100	32.44	67.56	25.91	74.09

#### The variables

We have 32,147 household-year observations. Here we give details of our key variables in the analysis:

##### Income

According to Equation (1), three types of income sources have to be estimated.

Firstly, household income (Y) is total earnings gained at the household level divided by the number of family members. The earnings include salaries paid by employers for the employed family members, retirement payments for the retired ones, self-gains for self-employed family members.^4^ Household income also include transfer payments from both governments and other private individuals. We sum up all these earnings to get total household income.

GTPs include all cash funds from the government that the household are able to get. The indicator is also in per capita form. If transfers from the government are in-kind benefits, the survey also asks “*how much money of these in-kind benefits value*”. Finally, we sum up GTPs of all family members within the household.

Similarly, PTPs are transfers either from employers, relatives or friends. PTPs are also measured at household-level, in the form of per capita.

##### Public services

From questionnaires, we have information about three kinds of public services provisions. They are medical insurance, clean water provision and residential hygiene protection. Equivalently, we have four dummy variables to measure public services provisions. The first dummy variable measures whether the householder has national primary medical insurance. The second dummy variable indicates whether the household have an access to clean water provision.^5^ The third dummy variable denotes whether the household have toilets inside their house, this indicator reflects public hygiene protection services. Similarly, the last dummy variable is also about hygiene conditions protected by municipal environment entities. It measures whether there are any excrement around the living place.

##### Poverty status

Poverty status is measured by a set of dummies:(1) The first poverty status dummy estimates whether the household’s income is below the average income level of their county. The household’s income is calculated as total household’s income divided by the number of family members. If the household’s income is below average county level, it equals to 1, otherwise it equals to 0. (2) The second poverty status dummy measures whether household’s income is less than county income-level at 25 quartile. The dummy equals to 1 for below and 0 for above.(3) Finally, we define the poverty status dummy according to family properties. If the household do not have a color television, the dummy variable equals to 1, otherwise 0.

##### Householder characteristics

We have a set of controlling variables of householder’s characteristics. They include gender(1 for male, 0 for female), age, marital status(1 for yes, 0 for 0), ethnicity(1 for minority, 0 for *Han* ethnicity), migrant status,^6^ educational level, employment status, occupation and a dummy variable measuring whether the householder has got a second job.

##### Households characteristics

Another set of controlling variables are used to measure characteristics at household-level. They include identity registration (1 for urban residents, 0 for rural residents), political connection(a dummy variable equals to 1 if any member from the household works in the government); a dummy variable equals to 1 if the household is identified as the “Five-Guarantee”.^7^ And the dummy variable indicates whether the household is a “one-child” family or not (1 for yes, 0 for no).

##### Family properties

Five kinds of family assets have been inferred in the survey. The respondent is asked whether to have a car, an air-conditioner, a camera, a washing machine or a fridge, respectively. The equivalent dummy variable equals to 1 for yes and 0 for no.

Several caveats to mention. On one hand, we choose price in 2006 as the benchmark to do price deflation. This enables us to compare present prices to previous ones. On the other hand, all variables are in *per capita* form. For example, poverty dummies are all constructed based on household income *per capita*. It ensures households of different sizes to be comparable.

Table 2 gives detailed definitions and summary statistics of all the variables used in the analysis.

**Table 2:** The Definitions and Summary Statistics

Variable	Definition of Variables	Mean	Std.Dev.	Obs
*Poverty_*_*1*_	1 for income below the county average, 0 for Not.	0.618	0.4858	32147
*Poverty_*_*50%*_	1 for income below the county median, 0 for Not.	0.4970	0.5000	32147
*Poverty_*_*25%*_	1 for income below the county 25% quartile, 0 for Not.	0.2457	0.4305	32147
*Poverty_*_*10%*_	1 for income below the county 10% quartile, 0 for No.	0.0954	0.2937	32147
*Col television*	1 for household not with a color television, 0 for Yes.	0.3985	0.4896	32147
*GTP* (100 yuan)	Average GTP obtained by the household	3.6594	17.075	32147
*PTP* (100 yuan)	Average PTP obtained by the household	5.1309	15.433	32119
*H_income*(100 yuan)	The household income per capita	49.054	79.292	32119
*Gender*	Dummy- is householder male or female	0.8442	0.3627	32147
*Age*	Age of the householder	50.4394	13.94	32011
*Minority*	Dummy-is householder a minority?	0.0267	0.1613	32147
*Migrant*	Dummy- is householder a migrant?	0.0209	0.1432	32147
*Secondedu*	Dummy- is householder graduated from high school?	0.1609	0.3674	32147
*Highedu*	Dummy-does householder get a bachelor degree(or higher)?	0.0362	0.1867	32147
*Job*	Dummy-does householder have a job?	0.7376	0.44	31732
*State*	Dummy-does householder work in public institutions?	0.235	0.424	32147
*Agriculture*	Dummy-does householder involve in agricultural activities?	0.1153	0.3193	32147
*Private*	Dummy-does householder work in private companies?	0.0664	0.249	32147
*Second-job*	Dummy-does householder have a second job?	0.098	0.3128	32147
*Urban*	Dummy- urban or rural identity?	0.3244	0.4682	32147
*Political*	Dummy- family members work in government?	0.0343	0.182	32147
*Fiveg*	Dummy- household belongs to “the five guarantees”?	0.002	0.0447	32147
*Single*	Dummy- household belongs to “one-child” family?	0.2591	0.4382	32147
*Insurance*	Dummy- does the householder have primary medical insurance?	0.3515	0.4775	32147
*Water*	Dummy-does the household have an access to clean drinking-water	0.6841	0.4649	31874
*Toilet*	Dummy- is there toilet inside the house?	0.3441	0.4751	32129
*Hygiene*	Dummy- is there any excrement around house?	0.8505	0.3565	32129
*Car*	Dummy- does the household own a car?	0.0283	0.1657	32129
*Air conditioner*	Dummy-does the household own an air-conditioner?	0.1072	0.3093	32129
*Camera*	Dummy-does the household own a camera?	0.1079	0.3103	32129
*Washer*	Dummy- does the household own a washing machine?	0.5077	0.4999	32129
*Fridge*	Dummy- does the household own a fridge?	0.3549	0.4785	32128

## Results

### The baseline results

Panel A in Table 3 represents the baseline results of SEM regressions. In model I, the poverty status is measured as whether the household’s income is below the average income level of their county. In model II, the poverty status is measured as whether household’s income is less than county income-level at 25 quartile. Column 1 to column 4 show results corresponding to Equation (1)–(4). Similarly, column 5 to column 8 also show results from Equation (1)–(4).

**Table 3:** Government Transfer Payments, Public Services and Poverty Status

Variables	Model I: Poverty = poverty__1_				Model II: Poverty = poverty__25%_			
	H_income	GTP	PTP	Poverty	H_income	GTP	PTP	Poverty
Panel A								
*H_ income*	—	0.1011^c^ (17.36)	0.0267^c^ (3.06)	−0.004^c^ (−12.84)	—	0.1013^c^ (17.39)	0.0223^c^ (2.56)	−0.0012^c^ (−3.75)
*GTP*	—	—	0.771^c^ (8.24)	0.013^c^ (5.03)	—	—	0.774^c^ (8.17)	0.0085^c^ (3.68)
*PTP*	—	—	—	−0.033^c^ (−12.77)	—	—	—	−0.026^c^ (−10.93)
*Job*	1996.32^c^ (15.32)	−497.10^c^ (−16.71)	212.35^c^ (4.77)	−0.0653^c^ (−4.21)	1989.56^c^ (15.27)	−497.46^c^ (−16.72)	225.62^c^ (5.06)	−0.016 (−1.13)
*Gender*	72.21 (0.6)	−103.35^c^ (−3.68)	−36.04 (−1.20)	0.0172 (1.63)	76.055 (0.63)	−103.50^c^ (−3.68)	−36.9 (−1.23)	0.00014 (0.01)
*Minority*	206.68 (0.78)	−6.14 (−0.10)	−46.84 (−0.73)	0.00471 (0.21)	205.31 (0.77)	−4.6096 (−0.07)	−48.34 (−0.76)	0.0065 (0.32)
*Urban*	510.35^c^ (4.59)	−265.44^c^ (−8.42)	136.15^c^ (3.99)	−0.0347^c^ (−2.81)	524.87^c^ (4.72)	−264.85^c^ (−8.43)	131.35^c^ (3.83)	−0.0106 (−0.94)
*Political*	−1368.42^c^ (−5.74)	86.214 (1.59)	89.09 (1.56)	−0.0870^c^ (−4.18)	−1361.34^c^ (−5.71)	86.1386 (1.59)	84.22 (1.47)	−0.0472^b^ (−2.49)
*Migrant*	−147.84 (−0.50)	63.961 (0.93)	238.21^c^ (3.36)	0.0301 (1.16)	−147.02 (−0.50)	64.102 (0.93)	237.72^c^ (3.35)	0.0266 (1.13)
*Age*	95.84^c^ (4.81)	−15.55^c^ (−3.33)	5.2356 (1.08)	−0.002 (−1.19)	95.01^c^ (4.77)	−15.61^c^ (−3.34)	5.845 (1.20)	−0.0048^c^ (−3.16)
*Age2*	−0.7232^c^ (−3.82)	0.1366^c^ (3.07)	−0.0564 (−1.22)	0.000016 (1.00)	−0.7203^c^ (−3.80)	0.1372^c^ (3.09)	−0.0598 (−1.29)	0.00005^c^ (3.15)
*Secondjob*	1124.22^c^ (8.30)	−141.04^c^ (−4.42)	−47.939 (−1.41)	−0.0759^c^ (−6.22)	1119.93^c^ (8.27)	−141.22^c^ (−4.42)	−39.02 (−1.15)	−0.1011^c^ (−9.11)
*Private*	508.46^c^ (2.87)		5.8603 (0.17)		517.74^c^ (2.93)		42.542 (1.28)	
*Agriculture*	−2008.66^c^ (−13.45)		202.81^c^ (5.67)		−1952.17^c^ (−13.10)		144.01^c^ (4.12)	
*State*	−410.03^c^ (−3.35)		419.54^c^ (15.31)		−431.75^c^ (−3.53)		435.43^c^ (16.01)	
*Secondedu*	1261.13^c^ (12.59)				1189.21^c^ (11.38)			
*Highedu*	4099.95^c^ (17.96)				4159.75^c^ (18.15)			
*Insurance*	1023.84^c^ (10.13)			−0.0313^c^ (−3.83)	1038.62^c^ (10.27)			−0.0560^c^ (−7.70)
*Water*	266.70^c^ (2.72)			−0.0298^c^ (−3.43)	266.22^b^ (2.71)			−0.0324^c^ (−4.14)
*Toilet*	1256.73^c^ (11.68)			−0.0214^b^ (−2.14)	1262.19^c^ (11.71)			−0.0256^c^ (−2.76)
*Hygiene*	327.59^c^ (3.06)			−0.0381^c^ (−4.59)	330.90^c^ (3.09)			−0.0476^c^ (−6.46)
*Fiveg*		59.07 (0.33)				−17.72 (−0.10)		
*Single*		−103.75^c^ (−3.75)				−105.19^c^ (−3.85)		
*Car*			243.79^c^ (4.23)				185.48^c^ (3.16)	
*Air conditioner*			155.28^c^ (2.74)				167.42^c^ (2.90)	
*_cons*	−2440.31^c^ (−3.94)	544.77^c^ (3.83)	35.736 (0.23)	1.0960^c^ (20.72)	−2408.40^c^ (−3.88)	546.61^c^ (3.84)	18.167 (0.12)	0.7383^c^ (15.37)
*Year*	Yes	Yes	Yes	Yes	Yes	Yes	Yes	Yes
*County*	Yes	Yes	Yes	Yes	Yes	Yes	Yes	Yes
Chi2()	7592.34^c^	3541.3^c^	3335.4^c^	2259.52^c^	7580.97^c^	3543.9^c^	3315.7^c^	1495.09^c^
obs	30628	30628	30628	30628	30628	30628	30628	30628
Panel B	Mechanisms							
Crowd-out Effect	−0.3875^c^ (−4.70)				−0.8739^c^ (−14.19)			
Inductive Effect	−2.705^c^ (−43.39)				−4.244^c^ (−27.61)			

Notes: To identify the mechanism, we introduce the interactive term between the poverty status and GTPs into PTP determination specification. And we also introduce he interactive term between the poverty status and GTPs into household-income determination specification. We only demonstrate results of the interactive term in Panel B. Other variables are not reported. Z values are reported in parentheses. ^a^Significantly at 10% level. ^b^Significantly at 5% level. ^c^Significantly at 1% level

As shown in column 2 as well as column 3, higher income-level household is associated with more transfer payments from both governments and other individuals. The coefficients of household income variable in GTP determination model and PTP determination model are both significantly positive. The results imply that GTPs and PTPs are essentially pro-rich. We are able to get similar results by estimating Model II.

The impacts of different anti-poverty policies on poverty reduction are presented in column4 and 8. We find that higher household income is associated with lower possibilities to become the poverty. Government transfer payments significantly increase the probabilities to become the poor, while private transfer payments are statistically negative related to the poverty status. The baseline results are partially consistent with previous literature. As suggested by [14], direct government transfers to the poor seems to have insignificant impact on poverty reduction in China.

Then we explain why GTP do not protect people from being the poverty. To examine the underlying mechanism, we firstly test the crowded-out effect between GTPs and PTPs. We introduce the interactive term between the poverty status and GTPs into Equation (3). We find that the interactive term is significantly negative. Economically, GTPs received by the poverty increase by 10%, PTPs decrease by 3.875%. The crowd-out effect is more pronounced among absolute low-income group, as the magnitude of the interactive term in Model II is larger than that of Model I.

We secondly examine the inductive effect of GTP on household income. Similarly, we introduce the interaction between GTPs and the poverty status into Equation (1). We find that the interactive term is also significantly negative. GTPs increase by 10%, the household income decrease by 2.705%. According to [37], GTPs make people more dependent on the external help from governments. They become more “lazy” instead of working hard, especially the low-skilled ones. We get similar results as we estimate the inductive effect in Model II.

We find that public services reduce the probabilities of being the poverty significantly, as can be seen from column 4 and column8. Meanwhile, we find that public services may reduce poverty through two mechanisms. Firstly, By providing some basic services, the government helps the poverty through sharing more living cost which are supposed to be taken by the poverty themselves. Secondly, it helps the poor gain higher household income and thus reduce the poverty indirectly.

The results for controlling variables are roughly similar to the previous literature. If the householder has got a job, then the entire household are more likely to have higher income level but receive less transfer payments from the government. Migrants do not obtain higher level of GTPs than native residents, however, they receive more PTPs actually. Householder’s age affects household’s income and GTPs in a non-linear way. On one hand, the household get more income and GTPs as the householder is becoming older. On the other hand, the magnitude of the age effect decreases as time goes on. The householder who has got a second job is associated with higher income level, however, entire family would not receive more PTPs from either employers or relatives. The household have the highest income level if the householder works in private companies. While the household gain less income if the householder works in agricultural sectors, governments or state-owned firms. However, the household obtain more PTPs when the householder works in agricultural sectors, governments and state-owned companies. Finally, educational background significantly increases household income.

People from urban areas gain higher household income and PTPs. Households in rural areas obtain more GTPs. Households that are partially related to governments have less income but more GTPs and PTPs. Surprisingly, “one-child” families have less transfers from governments. Households with considerate family properties obtain more PTPs. Overall, the results suggest that PTP is pro-rich, and the low-income group also benefit little from GTPs.

### GTP, public services and poverty status conversion

In this section, we examine whether GTPs or public services have significant impact on poverty status conversion. To test the relationship, we divide the sample into two groups. One group contains households who have seen no change in their poverty status, i.e., households remain the non-poverty or the poverty during the sample period. The other group contains households who have been changed at least once in terms of poverty status, i.e., from the non-poverty status to the poverty status, or from the poverty status to the non-poverty status. In the poverty status conversion group, we require each household must at least have two observations from different wave of survey. The poverty status is also defined as we do in Table 3. The results are presented in Table 4.

**Table 4:** Government Transfer Payments, Public Services and Poverty Status Conversion

Variable	Poverty = poverty__1_				Poverty = poverty__25%_			
	Change in Poverty		No Change in Poverty		Change in Poverty		No Change in Poverty	
	H_income	Poverty	H_income	Poverty	H_income	Poverty	H_income	Poverty
Panel A								
*H_income*		−0.008^c^ (−6.11)		−0.004^c^ (−12.35)		−0.006^b^ (−2.54)		−8.34e-04^c^ (−2.89)
*GTP*		0.0215^c^ (5.08)		0.0061^b^ (1.97)		0.012^a^ (1.90)		0.010^c^ (4.58)
*PTP*		−0.012^c^ (−2.40)		−0.035^c^ (−13.23)		−0.016^b^ (−2.14)		−0.023^c^ (−10.34)
*Insurance*	264.78 (1.47)	−0.0022 (−1.14)	1213.09^c^ (10.22)	−0.0483^c^ (−5.21)	103.46 (0.74)	−0.0316^b^ (−1.98)	1144.37^c^ (9.68)	−0.0447^c^ (−6.15)
*Water*	192.79 (1.22)	−0.0136 (−0.94)	255.69^b^ (2.20)	−0.0429^c^ (−4.20)	280.14^b^ (2.22)	−0.0186^b^ (−2.02)	281.97^b^ (2.39)	−0.0439^c^ (−5.48)
*Toilet*	544.33^c^ (3.05)	−0.0148^a^ (−1.90)	1529.19^c^ (12.14)	−0.0214^a^ (−1.78)	310.41^b^ (2.15)	−0.0017 (−1.09)	1282.63^c^ (10.27)	−0.0319^c^ (−3.42)
*Hygiene*	61.44 (1.35)	−0.0108 (1.63)	392.50^c^ (3.13)	−0.0435^c^ (−4.62)	36.95 (1.28)	−0.0054 (−1.31)	384.27^c^ (2.98)	−0.0588^c^ (−7.92)
*Other controls*	Yes	Yes	Yes	Yes	Yes	Yes	Yes	Yes
Year	Yes	Yes	Yes	Yes	Yes	Yes	Yes	Yes
County	Yes	Yes	Yes	Yes	Yes	Yes	Yes	Yes
Chi2()	6869.8^c^	7499.35^c^	5927.02^c^	2674.41^c^	735.65^c^	7119.2^c^	7128.01^c^	1340.81^c^
obs	8468	8468	22160	22160	7121	7121	23507	23507
Panel B	Mechanisms							
Crowd-out Effect	−0.1461 (−0.67)		−0.5163^c^ (−4.76)		0.0129 (0.04)		−1.0567^c^ (−15.63)	
Inductive Effect	−2.4895^c^ (−19.28)		−2.7305^c^ (−38.35)		−2.6975^c^ (−12.61)		−5.1667^c^ (−24.18)	

Note: We only report the results for household-income determination specification and the poverty-status determination specification in Table 4. ^a^Significantly at 10% level.^b^Significantly at 5% level.^c^Significantly at 1% level

The results show that household income as well as PTPs have negative effect on poverty status conversion. In other words, they prevent the non-poverty people from being the poverty again. While the GTPs have a significant impact on promoting the non-poverty into the poverty. Public services significantly reduce the incidence of poverty status conversion. By examining mechanisms, the crowd-out effect and the inductive effect still hold. Transfers from governments make the poverty receive less PTPs and become more dependent on external assistance. Moreover, these two effects are more pronounced among the relatively poor group(by using the poverty__25%_ indicator). Other controlling variables remain largely unchanged compared to Table 3.

### Heterogeneity test

In this section, we have a heterogeneity test. In China, situations in urban areas and rural areas are quite different. For instance, very few rural residents have formal jobs. Neither do they have as many financial resources as urban residents do. There are also essential differences in the way of the GTP allocation process for these two groups. Being lack of strict management, GTPs in rural areas could be more likely to be misused. We divide the sample into two groups. One group contains households in urban areas while the other contains households from rural areas. We re-run SEM models and the results are shown in Table 5.

**Table 5:** Heterogeneity between urban and rural households (Poverty = poverty__1_)

Variable	(1)Urban		(2) Urban Natives		(3) Rural		(4) Rural Natives	
	H_income	Poverty	H_income	Poverty	H_income	Poverty	H_income	Poverty
*Panel A*								
*H_income*		−0.004^c^ (−8.91)		−0.0039^c^ (−8.69)		−0.0051^c^ (−10.66)		−0.005^c^ (−10.68)
*GTP*		−2.27E-05 (−0.01)		1.20 e-04 (0.07)		0.035^c^ (5.32)		0.038^c^ (5.64)
*PTP*		−0.020^c^ (−6.16)		−0.020^c^ (−6.20)		−0.046^c^ (−10.00)		−0.046^c^ (−9.89)
*Insurance*	1568.53^c^ (9.1)	−0.042^c^ (−3.24)	1581.67^c^ (8.87)	−0.044^c^ (−3.37)	914.01^c^ (6.94)	−0.024^b^ (−2.06)	907.38^c^ (6.89)	−0.026^b^ (−2.17)
*Water*	107.32 (1.42)	−0.0091 (−0.55)	135.54 (0.52)	−0.0107 (−0.64)	572.09^c^ (5.46)	−0.0145 (−1.34)	584.03^c^ (5.57)	−0.0142 (−1.30)
*Toilet*	1102.48^c^ (6.43)	−0.0398^c^ (−3.04)	1151.58^c^ (6.48)	−0.0437^c^ (−3.29)	1271.71^c^ (9.5)	−0.0134 (−1.04)	1215.51^b^ (9.08)	−0.0178 (−1.38)
*Hygiene*	443.00^a^ (1.7)	−0.033^a^ (−1.90)	439.46^a^ (1.64)	−0.036^b^ (−2.06)	439.37^c^ (3.65)	−0.027^b^ (−2.52)	439.51^c^ (3.66)	−0.025^b^ (−2.38)
Controls	Yes	Yes	Yes	Yes	Yes	Yes	Yes	Yes
Year	Yes	Yes	Yes	Yes	Yes	Yes	Yes	Yes
County	Yes	Yes	Yes	Yes	Yes	Yes	Yes	Yes
Chi2()	3871.2^c^	1333.4^c^	3662.9^c^	1223.1^c^	11042.7^c^	18934.7^c^	10968.6^c^	18460.5^c^
obs	9805	9805	9367	9367	20823	20823	20605	20605
*Panel B*	Mechanisms							
Crowd-out Effect	−0.3934^c^ (−5.37)		−0.3969^c^ (−5.54)		−0.6289^c^ (−4.09)		−0.6094^c^ (−3.96)	
Inductive Effect	−2.1650^c^ (−26.41)		−2.1657^c^ (−26.07)		−3.4267^c^ (−38.03)		−3.4167^c^ (−37.99)	

Note: ^a^Significantly at 10% level. ^b^Significantly at 5% level. ^c^Significantly at 1% level

For urban residents, no matter they are migrants or natives, GTPs have no significant impact on reducing the poverty incidence. For rural residents, GTPs prevent the poverty from being out of poverty trap. However, almost all public services significantly reduce the poverty incidence. By checking the mechanisms, the “crowd-out ” effect as well as the “inductive” effect holds whatever sub-sample we estimate. These two effects, however, are more pronounced among relatively lower-income level group in rural areas.

### The impact on the absolute poverty

The above analysis measures the poverty from relative perspective. But considerate people are labeled as the absolute poverty in China, as they are totally disabled or have been caught in serious illness [10]. In order to estimate the impact of GTPs (public services) on the absolute poverty status, we focus on the absolute poverty sub-group. We only keep households who make livings mainly on transfer payments(including GTPs and PTPs) or government subsidies. When household receive more transfers or subsidies than any other kinds of income sources, the household is regarded as the absolute poverty. As transfers are allocated on the basis of more strict regulation, we further use the sub-sample who are mainly dependent on transfers. Results are shown in Table 6.

**Table 6:** The Effects of GTP and Public Services on the Absolute Poverty

Variable	Poverty = poverty__1_				Poverty = poverty__25%_			
	Transfer/Subsidy as main income		Transfer as main income		Transfer/Subsidy as main income		Transfer as main income	
	H_income	Poverty	H_income	Poverty	H_income	Poverty	H_income	Poverty
*Panel A*								
*H_income*		−0.00343^a^ (−1.81)		−0.00123^a^ (−1.86)		−0.0037^a^ (−1.68)		−3.14E-04 (−1.19)
*GTP*		−0.0011 (−0.83)		−0.00184 (−1.48)		0.0029^b^ (2.00)		−6.26E-04 (−0.42)
*PTP*		−0.010^c^ (−4.91)		−0.0095^c^ (−5.48)		−0.008^c^ (−3.40)		−0.006^c^ (−2.79)
*Insurance*	772.79^c^ (6.76)	−0.01296 (−0.64)	811.30^c^ (6.28)	−0.0322 (−1.60)	785.32^c^ (6.86)	−0.0179 (−0.77)	809.33^c^ (6.27)	−0.0368 (−1.51)
*Water*	−17.57 (−0.15)	−0.0350^b^ (−1.98)	54.0507 (0.41)	−0.00574 (−0.31)	−13.0575 (−0.11)	−0.040^a^ (−1.94)	56.0181 (0.42)	−0.0242 (−1.08)
*Toilet*	516.59^c^ (4.59)	−0.0345^a^ (−1.69)	608.89^c^ (4.77)	−0.03536^a^ (−1.65)	520.84^c^ (4.62)	−0.0201 (−0.90)	612.05^c^ (4.80)	−0.0229 (−0.90)
*Hygiene*	28.3617 (1.22)	−0.0396^b^ (−2.03)	−12.179 (−0.09)	−0.0458^b^ (−2.33)	28.4244 (1.22)	−0.074^c^ (−3.24)	−12.998 (−0.09)	−0.098^c^ (−4.09)
Controls	Yes	Yes	Yes	Yes	Yes	Yes	Yes	Yes
Year	Yes	Yes	Yes	Yes	Yes	Yes	Yes	Yes
County	Yes	Yes	Yes	Yes	Yes	Yes	Yes	Yes
Chi2()	1178.3^c^	837.60^c^	1186.22^c^	879.03^c^	1177.3^c^	832.85^c^	1189.7^c^	764.90^c^
obs	3693	3693	3069	3069	3693	3693	3069	3069
*Panel B*	Mechanisms							
Crowd-out Effect	−0.0268 (−0.59)		0.0421 (1.05)		0.5774^c^ (2.92)		0.4736^c^ (2.7)	
Inductive Effect	−0.3617^c^ (−12.32)		−0.3739^c^ (−11.74)		−0.9695^c^ (−14.73)		−0.9962^c^ (−14.31)	

Note: ^a^Significantly at 10% level.^b^Significantly at 5% level.^c^Significantly at 1% level

The results show some interesting findings. The GTPs have insignificant impact on reducing the absolute poverty incidence. But they do not encourage the absolute poverty incidence neither. While public services still significantly reduce the absolute poverty rate. The “crowd-out” effect and “inductive” effect of the GTPs on the absolute poverty are estimated. Surprisingly, the interactive term between GTPs and PTPs is significantly positive, implying that GTPs lead to more PTPs. This may be the demonstration effect generated by government transfers. When the government increases its public transfers to help the absolute poor, it also encourage other social members to help them together. Whereas, GTPs still has negative impact on enhancing the household income, considering about the inductive effect.

## Discussion

### Robustness checks

In this section, we have two robustness checks: On one hand, we change the measurement of poverty status. On the other hand, we change model specifications.

#### Change the measurement of poverty status

In first robustness check, we use three other measures of poverty status. The first poverty status measures whether household’s income is less than county median income-level(1 for yes, 0 for no). The second measures whether household’s income is below county income-level at 10 quartile(1 for yes, 0 for no). The final one measures whether the household have the most basic family property, i.e., TV. The results are presented in Table 7.

**Table 7:** Robustness Check: Other Poverty Status Measurement

Variable	Poverty = poverty__50_		Poverty = poverty__10_		Poverty = no television	
	H_income	Poverty	H_income	Poverty	H_income	Poverty
*Panel A*						
*H_income*		−0.0037^c^ (−9.88)		−1.55e-04^a^ (−1.95)		−1.38e-04^b^ (−2.09)
*GTP*		0.0108^c^ (4.08)		0.002^c^ (3.2)		0.025^c^ (12.41)
*PTP*		−0.035^c^ (−12.53)		−0.014^c^ (−8.73)		−0.017^c^ (−8.14)
*Insurance*	1023.85^c^ (10.13)	−0.0381^c^ (−4.49)	1032.29^c^ (10.2)	−0.0274^c^ (−5.55)	1063.08^c^ (10.51)	−0.05879^c^ (−9.59)
*Water*	262.22^c^ (2.67)	−0.0283^c^ (−3.12)	273.72^b^ (2.79)	−0.01715^c^ (−3.26)	281.53^c^ (2.87)	−0.1067^c^ (−16.14)
*Toilet*	1245.10^c^ (11.57)	−0.0194^b^ (−1.84)	1266.67^c^ (11.72)	−0.00915 (−1.49)	1325.84^c^ (12.33)	−0.1341^c^ (−17.04)
*Hygiene*	327.84^c^ (3.06)	−0.0507^c^ (−5.88)	332.09^c^ (3.1)	−0.0274^c^ (−5.45)	332.38^c^ (3.1)	−0.0572^c^ (−9.24)
Controls	Yes	Yes	Yes	Yes	Yes	Yes
Year	Yes	Yes	Yes	Yes	Yes	Yes
County	Yes	Yes	Yes	Yes	Yes	Yes
Chi2()	7587.86^c^	2013.96^c^	7576.25^c^	777.65^c^	7649.84^c^	16897.42^c^
obs	30628	30628	30628	30628	30628	30628
*Panle B*	Mechanisms					
Crowd-out Effect	−0.5943^c^ (−9.18)		−0.7250^c^ (−7.28)		5.7516^c^ (4.69)	
Inductive Effect	−2.9593^c^ (−33.11)		−9.7151^c^ (−28.22)		−2.2881^c^ (−112.32)	

Note: ^a^Significantly at 10% level.^b^Significantly at 5% level.^c^Significantly at 1% level

As shown in Table 7, our results remain largely unchanged. Government transfer payments have significantly negative effect on poverty protection. GTPs crowd out private transfer payments received by the household and make the poverty more dependent on the government. Whereas, public services have significantly positive effect on poverty reductions. Public services help the poverty gain higher household income and share living cost which are supposed to be taken by the poor themselves.

#### Adjustment of model specifications

In baseline regressions, both GTPs and PTPs are not affected by poverty status. However, it may be the case that the poverty are able to gain more (or less) GTPs and PTPs. Therefore, in the second robustness check, we introduce poverty status variable into different sub-specifications: household income determination equation, GTP determination equation and PTP determination equation. We re-estimate the simultaneity equation models and results are reported in Table 8.

**Table 8:** Robustness Check: Change Model Specification

Variable	Introduce poverty status in GTP model				Introduce poverty status in GTP and PTP model			
	H_income	GTP	PTP	Poverty	H_income	GTP	PTP	Poverty
*Panel A*								
*H_income*		0.1201^c^ (10.57)	0.0274^c^ (3.14)	−0.0046^c^ (−13.12)		0.1235^c^ (10.84)	−0.0244 (−1.23)	−0.0047^c^ (−13.74)
*Poverty*		277.04^a^ (1.83)				328.90^b^ (2.17)	−1116.8^c^ (−3.82)	
*GTP*			0.7783^c^ (8.3)	0.013^c^ (5.23)			0.4482^c^ (5.11)	0.0134^c^ (5.63)
*PTP*				−0.032^c^ (−12.22)				−0.034^c^ (−13.33)
*Insurance*	1029.67^c^ (10.21)			−0.0307^c^ (−3.77)	1038.55^c^ (9.54)			−0.0178^c^ (−3.10)
*Water*	293.44^c^ (2.98)			−0.0303^c^ (−3.50)	152.82 (1.43)			−0.0272^c^ (−3.46)
*Toilet*	1273.93^c^ (11.85)			−0.0212^b^ (−2.13)	1330.35^c^ (11.93)			−0.0156^b^ (−1.96)
*Hygiene*	348.51^c^ (3.24)			−0.0382^c^ (−4.61)	251.74^b^ (2.14)			−0.0273^c^ (−3.88)
Controls	Yes	Yes	Yes	Yes	Yes	Yes	Yes	Yes
Year	Yes	Yes	Yes	Yes	Yes	Yes	Yes	Yes
County	Yes	Yes	Yes	Yes	Yes	Yes	Yes	Yes
Chi2()	7590.53^c^	3512.1^c^	3333.9^c^	2227.98^c^	7579.52^c^	3509.7^c^	3916.7^c^	2262.28^c^
obs	30628	30628	30628	30628	30628	30628	30628	30628
*Panle B*	Mechanisms							
Crowd-out Effect	−0.3822^c^ (−4.54)				−0.1674^c^ (−2.60)			
Inductive Effect	−2.6715^c^ (−42.66)				−2.6535^c^ (−42.28)			

Notes: We use the poverty_1_ variable to measure the poverty status. ^a^Significantly at 10% level.^b^Significantly at 5% level.^c^Significantly at 1% level

As shown in Table 8, GTPs fail to protect the poverty in China. While public services reduce poverty incidence significantly. The crowd out effect and inductive effect still hold.

### Policy selection: from GTP to public services

We have empirically examined the impact of both GTP and public services on poverty reductions. Meanwhile, we test the interrelationships among GTP, PTP and public services. GTPs fail to protect the poor while public services are conducive to help the poverty out of poverty trap. Results are robust after considering about the heterogeneity and different model specifications.

GTPs mainly aim to help specific poor groups. These cash funds are initially given by the central government, while it is ultimately managed by local governments. Usually, GTPs have to be transferred through multiple layers of governments before they reach to the poverty. Each level of government does have strong incentives to misuse these funds for other economic purposes, such as infrastructure. It is also well known that the management of GTP is not with strict supervision. High income-level people, in some cases, are usually mistaken as the target group. Although GTP do have positive impact on reducing the absolute poverty, they crowd out the amount of PTP and make people more dependent on external sources.

However, public services essentially reduce poverty incidence. On one hand, they directly share the living cost which are supposed to be taken by the poverty. On the other hand, they help the poverty enhance earning abilities to gain higher household income. Moreover, targeting errors can be well prevented, as public services are equally provided to all the residents. In reality, the poor benefit from basic public services as much as the rich do. In summary, it is more optional for governments to choose public services as main anti-poverty policy.

## Conclusion

In this paper, we empirically test the effects of different anti-poverty policies on poverty reduction. We use eight waves of CHNS survey data, and apply with SEM to do the estimation. We find that GTPs are pro-rich, i.e., people with higher income obtain more government transfer payments. Meanwhile, GTPs crowd out PTPs that the poverty can receive and make them more dependent on external help. These effect are more pronounced among relatively low-income level group. However, public services significantly reduce poverty incidence and help to increase the poverty’s earning abilities. The effects are robust after the inclusion of other control variables as well as estimating with different specifications.

Anti-poverty has always been an concerning issue. To fight against poverty problems, many anti-poverty tools have been provided. As some of the policies cost a substantial amount of fiscal budget, whether they remain effective in reducing the poverty incidence matters a lot to governments. According to this study, governments directly offering cash to the poverty is not effective in reducing poverty incidence, whereas, public services are suggested to be adopted by governments to help the poor out of poverty trap.

## Abbreviations

CHNS: China Health and Nutrition Survey
GTP: Government transfer payments
PTP: Private transfer payments
SEM: Simultaneity equation model

## Footnotes

1 Cash transfers to people who have been totally or partially lost their abilities to work. The amount of grants to people who are eligible for this scheme depends on the local budget.

2 The minimum living cost standard is made by the national government. The amount of subsidy standard differs from city to city. Actually, it depends on the fiscal budget of each city. However, the bottom line is to make sure applicants at least are able to catch up with the national minimum living cost.

3 Each household has a code and each respondent has been required to document his(her) family code.

4 If the respondent is a businessman, his household income is his earnings from business involvements. If he is a peasant, household income is gains from agriculture activities.

5 Actually, in China, most urban residents have access to use clean water resources provided by municipal drinking-water companies. While many rural residents still have to dig water from underground by themselves.

6 In the questionnaire, there are two ways to identify the migrant status. Item A16 asks the respondent “whether you have always been living here. A21 asks the respondent“what is your home province”. We compare the respondent’s host province with his home province, and we are able to identify whether he is a migrant or not. By adopting this method, we reduce the possibilities when the respondent responses at will.

7 If the household is identified as the “Five-Guarantee”, it means the commune will take care of this family in terms of food consumption, clothing, medical care, housing and burial expenses.
